# Stress hormones promote DNA damage in human oral keratinocytes

**DOI:** 10.1038/s41598-021-99224-w

**Published:** 2021-10-05

**Authors:** Vitor Bonetti Valente, Diovana de Melo Cardoso, Giseli Mitsuy Kayahara, Giovana Barros Nunes, Kellen Cristine Tjioe, Éder Ricardo Biasoli, Glauco Issamu Miyahara, Sandra Helena Penha Oliveira, Gisele Zoccal Mingoti, Daniel Galera Bernabé

**Affiliations:** 1 Psychoneuroimmunology Laboratory, Psychosomatic Research Center, Oral Oncology Center, School of Dentistry, São Paulo State University (Unesp), 1193 José Bonifácio St, Araçatuba, São Paulo 15050-015 Brazil; 2 Department of Diagnosis and Surgery, School of Dentistry, São Paulo State University (Unesp), 1193 José Bonifácio St, Araçatuba, São Paulo 15050-015 Brazil; 3 Laboratory of Reproductive Physiology, Department of Animal Health, School of Veterinary Medicine, São Paulo State University (Unesp), 793 Clovis Pestana St, Araçatuba, São Paulo 16050-680 Brazil; 4 Laboratory of Immunopharmacology, Department of Basic Sciences, School of Dentistry, São Paulo State University (Unesp), 1193 José Bonifácio St, Araçatuba, São Paulo 15050-015 Brazil

**Keywords:** Cancer, Head and neck cancer, Oral cancer, Cancer, Cell biology

## Abstract

Chronic stress increases the systemic levels of stress hormones norepinephrine and cortisol. As well as tobacco-specific carcinogen NNK (4-(methylnitrosamine)-1-(3-pyridyl)-1-butanone), they can induce expressive DNA damage contributing to the cancer development. However, it is unknown whether stress hormones have genotoxic effects in oral keratinocytes. This study investigated the effects of stress hormones on DNA damage in a human oral keratinocyte cell line (NOK-SI). NOK-SI cells stimulated with norepinephrine or cortisol showed higher DNA damage compared to untreated cells. Norepinephrine-induced DNA damage was reversed by pre-treatment with beta-adrenergic blocker propranolol. Cells treated with NNK combined to norepinephrine displayed reduced levels of caspases 3 and 7. Cortisol also reduced the activity of pro-apoptotic enzymes. NNK or norepinephrine promoted single-strand breaks and alkali-label side breaks in the DNA of NOK-SI cells. Pre-treatment of cells with propranolol abolished these effects. Carcinogen NNK in the presence or absence of cortisol also induced DNA damage of these cells. The genotoxic effects of cortisol alone and hormone combined with NNK were blocked partially and totally, respectively, by the glucocorticoid receptor antagonist RU486. DNA damage promoted by NNK or cortisol and carcinogen combined to the hormone led to intracellular γH2AX accumulation. The effects caused by NNK and cortisol were reversed by propranolol and glucocorticoid receptor antagonist RU486, respectively. Propranolol inhibited the oxidation of basis induced by NNK in the presence of DNA-formamidopyrimidine glycosylase. DNA breaks induced by norepinephrine in the presence or absence of NNK resulted in higher 8OHdG cellular levels. This effect was also induced through beta-adrenergic receptors. Together, these findings indicate that stress hormones induce DNA damage of oral keratinocytes and could contribute to oral carcinogenesis.

## Introduction

Psychological stress activates the Sympathetic Nervous System (SNS) and the Hypothalamic–Pituitary–Adrenal (HPA) axis up-regulating the circulating levels of stress hormones^1^. The activation of SNS increases the production of catecholamines norepinephrine and epinephrine while HPA axis induces the cortisol secretion^1^. Exposure to these hormones released from chronic stress response has been associated to an enhanced risk of developing diseases such as cancer^1,2^. In somatic cells, norepinephrine and cortisol may induce DNA damage through the activation of beta-adrenergic and glucocorticoid receptors, respectively^3,4^. Both mechanisms trigger similar effects to those produced by the tobacco smoke carcinogens^5,6^. A significant damage in the integrity of DNA unleashed by these substances may lead to genome mutations and affect oncogenic mechanisms predisposing to cell malignant transformation^2,5^.

Chronic stress and stress hormones may cause a significant DNA damage accompanied by a higher production of phosphorylated histone H2AX (γH2AX)^7,8^ and 8-hydroxy-2′-deoxyguanosine (8OHdG)^2,9^. Both molecules are considered mutagenic biomarkers of the DNA damage and significantly enhance the occurrence of tumorigenic mutations into the genome of somatic cells, which may become malignant^2,10^. Moreover, stress hormones can inhibit the apoptosis of somatic cells^11,12^ allowing their replication with DNA damage. This inhibition is also considered another crucial mechanism related to the acquisition of malignant phenotype^2,3^. The activity of caspases is measured to assess cell apoptosis and may become downregulated after chronic exposure to the stress hormones and carcinogenic agents^13,14^.

Oral cancer represents one of the most common malignancies worldwide and its occurrence has been widely associated to the tobacco smoking^15^. In addition, chronic stress and stress hormones have also been investigated in oral cancer patients and preclinical models of the disease^16–20^. We recently demonstrated that biobehavioral factors are related to increased circulating norepinephrine levels in these patients^16^. In an orthotopic model, chronic stress up-regulated the plasma levels of catecholamines and glucocorticoids, which would contribute to increase tumor size and invasiveness^17^. In rats underwent chemical carcinogenesis, we showed that the stress hormones levels in the normal microenvironment predict the risk of developing oral cancer^18^. An expressive DNA damage caused by the chemical substances such as nicotine and tobacco-specific nitrosamines may be considered one of the first molecular events for oral cancer occurrence^5^. However, it remains unknown whether stress hormones have genotoxic effects in oral epithelial cells.

In the current study, we tested the hypothesis that the chronic exposure of an oral keratinocyte cell line (NOK-SI) to the stress hormones in the presence or absence of chemical carcinogen NNK (4-(methylnitrosamine)-1-(3-pyridyl)-1-butanone), a tobacco-specific nitrosamine, would induce DNA damage. In addition, these cells were also evaluated for the production of molecules associated to DNA damage and apoptosis.

## Results

### Norepinephrine and cortisol induce DNA damage in oral epithelial cells

**Figure 1:**
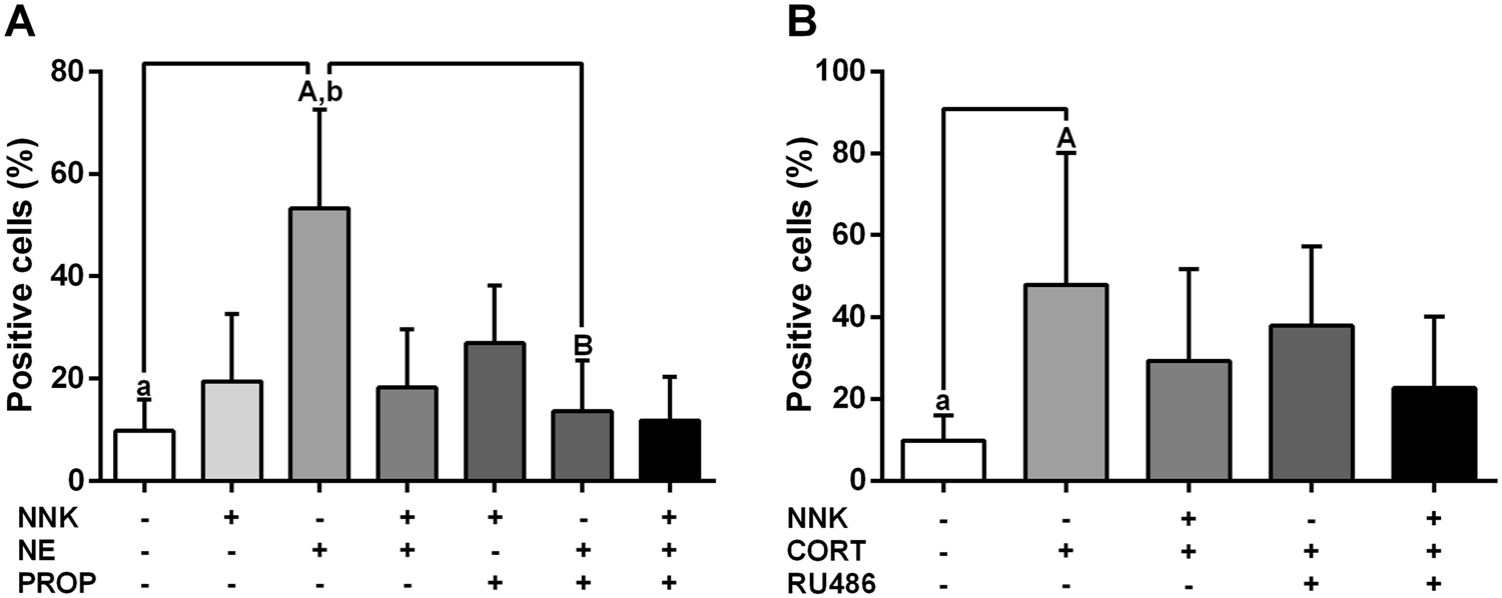
Stress hormones promote DNA damage in oral keratinocytes. (**A**) Norepinephrine increased the DNA damage in oral epithelial cells and this effect was abolished by the non-selective beta-blocker propranolol. (**B**) Cortisol increased the DNA fragmentation of oral keratinocytes. The results are expressed as the mean ± standard deviation (SD). Upper- and lower cases equal letters indicate a statistically significant difference (*p* < 0.05). NNK = 4 (N-metil-N-nitrosamine)-1-(3-piridil)-butano-1-one). NE = norepinephrine. PROP = propranolol. CORT = cortisol. NNK, NE, PROP and RU486 were used at 10 µM. CORT was used at 100 nM. NOK-SI cells were exposed to NNK and/or stress hormones in the presence or absence of their antagonists for 72 h.

TUNEL assay was performed to evaluate whether carcinogen NNK and stress hormones would promote a severe DNA fragmentation at the terminal deoxynucleotidyl transferase dUTP nick-end in NOK-SI cells. After 72 h of treatment, NOK-SI cells stimulated with norepinephrine displayed higher percentage of positive-TUNEL cells than untreated group (*p* < 0.0001; Fig. 1A). Norepinephrine-induced DNA damage was blocked by the pre-treatment with nonselective beta-blocker propranolol (*p* < 0.0001; Fig. 1A). Cells stimulated with cortisol showed an increase of the DNA damage when compared to control cells (*p* = 0.0004; Fig. 1B). This effect was partially abolished by the glucocorticoid receptor antagonist RU486 (*p* > 0.05; Fig. 1B). NNK treatment did not induced a significant DNA fragmentation when compared to the untreated group (*p* > 0.05; Fig. 1A). However, stimulation with carcinogen NNK inhibited the genotoxic effect induced by the stress hormones (*p* > 0.05; Fig. 1A, B).

### Cortisol inhibits the activity of apoptotic enzymes in oral keratinocytes

**Figure 2:**
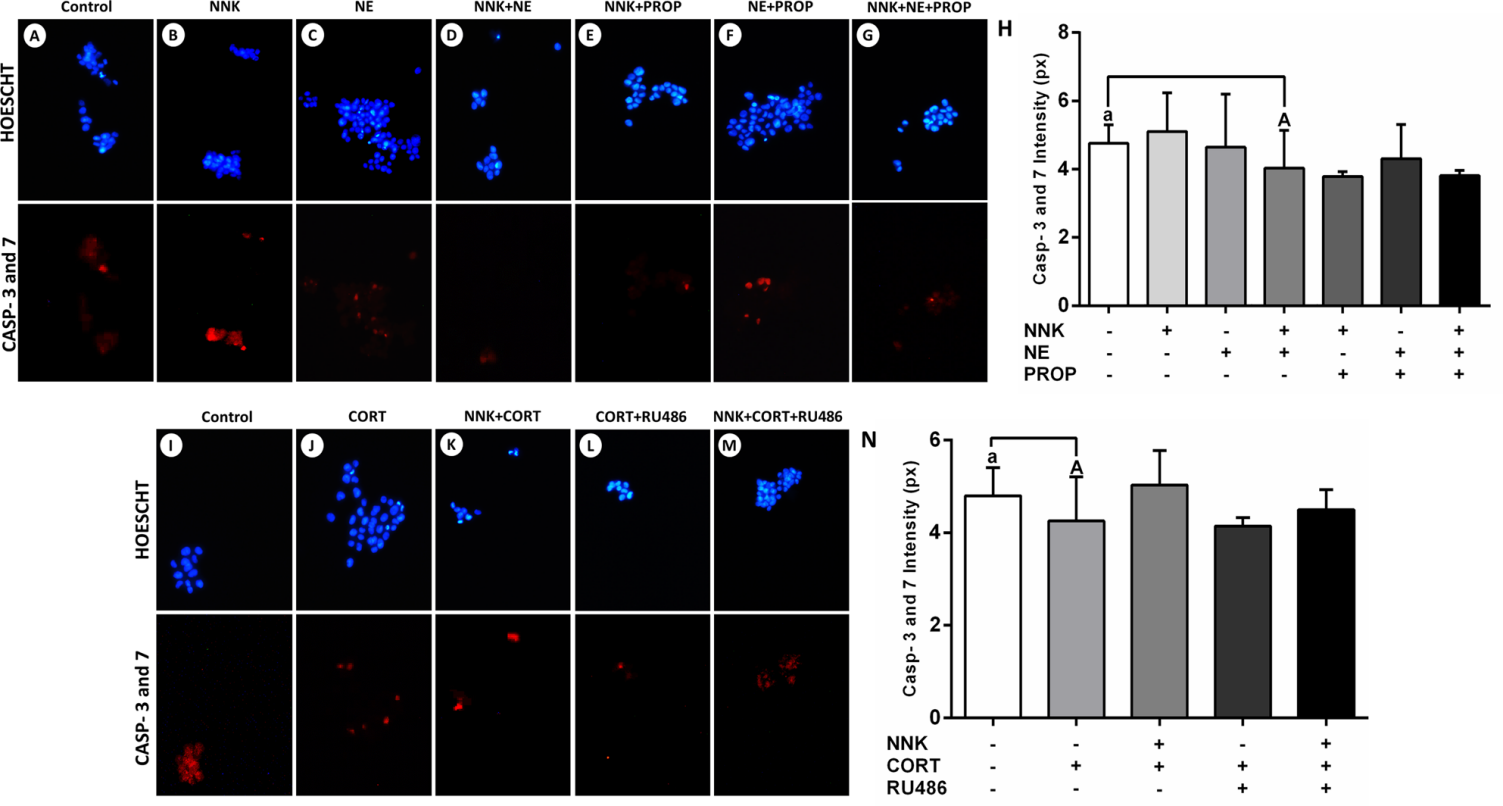
Effects of tobacco-specific nitrosamine NNK and norepinephrine on the activity of the caspases 3 and 7. (**A**) Control. (**B**) NNK. (**C**) Norepinephrine. (**D**) NNK and norepinephrine. (**E**) NNK and propranolol. (**F**) Norepinephrine and propranolol. (**G**) NNK, norepinephrine and propranolol. (**H**) Combined treatment of NNK with norepinephrine inhibited the activity of the pro-apoptotic enzymes. This effect was not abolished by the beta-adrenergic blocker propranolol. Cortisol inhibits the activity of caspases 3 and 7. (**I**) Control. (**J**) Cortisol. (**K**) NNK and cortisol. (**L**) Cortisol and RU486. (**M**) NNK, cortisol and RU486. (**N**) Cortisol inhibited the activity of pro-apoptotic enzymes. This effect was not abolished by the glucocorticoid receptor blocker RU486. The results are expressed as the mean ± standard deviation (SD). Upper- and lower cases equal letters indicate a statistically significant difference (*p* < 0.0001). NNK = 4 (N-metil-N-nitrosamine)-1-(3-piridil)-butano-1-one). NE = norepinephrine. PROP = propranolol. CORT = cortisol. NNK, NE, PROP and RU486 were used at 10 µM. CORT was used at 100 nM. NOK-SI cells were exposed to NNK and/or stress hormones in the presence or absence of their antagonists for 72 h.

The effects of NNK and stress hormones on the activity of caspases 3 and 7 were measured in NOK-SI cells. Carcinogen NNK or norepinephrine did not change the levels of caspases concerning the untreated group (*p* > 0.05; Fig. 2B, C, H). Nevertheless, cells treated with NNK combined to the norepinephrine showed lower levels of the apoptotic enzymes (*p* < 0.0001; Fig. 2D, H). Cells treated with cortisol displayed lower levels of caspases 3 and 7 than untreated cells (*p* < 0.0001; Fig. 2J, N). However, this effect was not inhibited by the glucocorticoid receptor antagonist RU486 (*p* > 0.05; Fig. 2L, N).

### Stress hormones promote single-strand breaks and alkali-label side breaks in the DNA of oral epithelial cells

**Figure 3:**
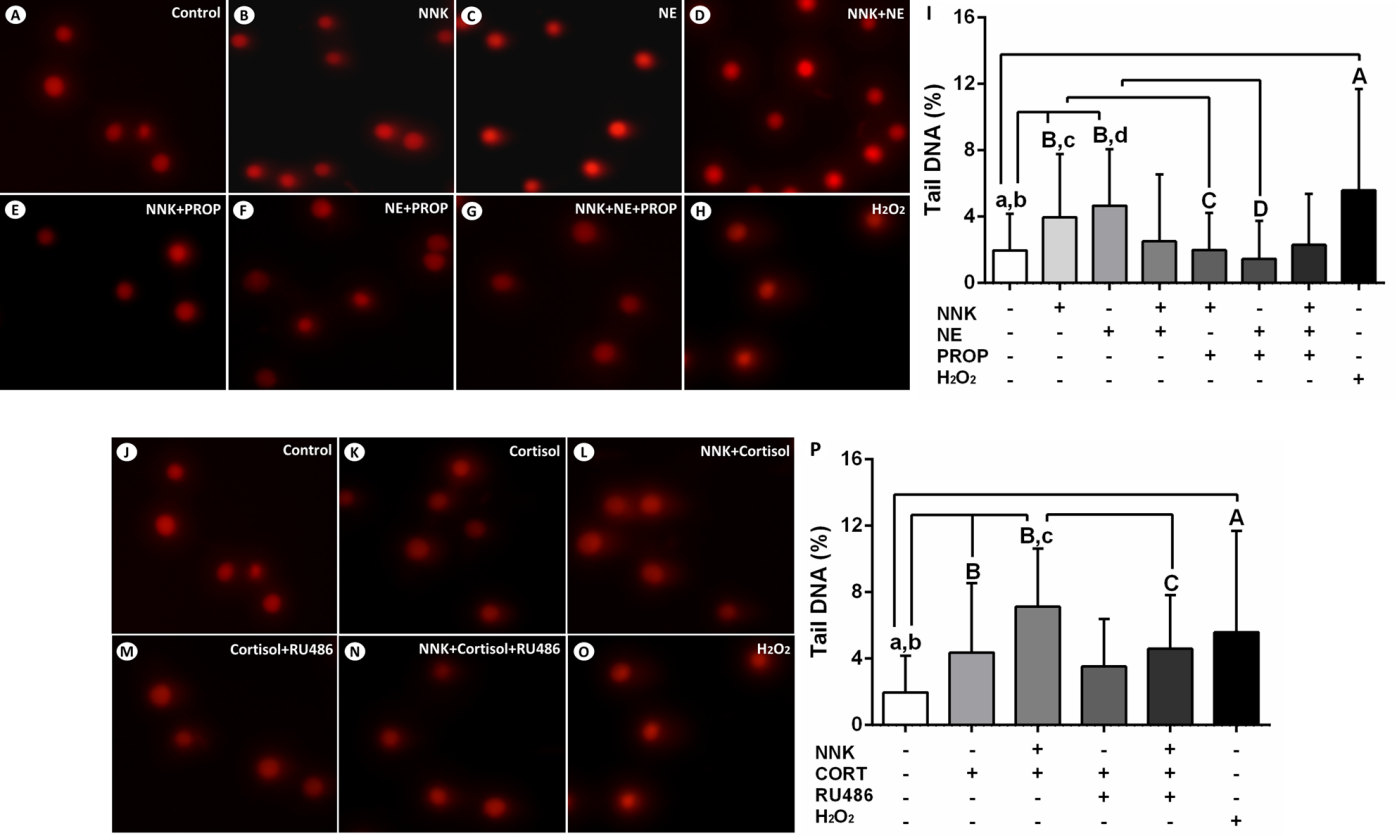
Stress hormones and NNK induce single-strand breaks and alkali-label side breaks in the DNA of oral epithelial cells. (**A**) Control. (**B**) NNK. (**C**) Norepinephrine. (**D**) NNK and norepinephrine. (**E**) NNK and propranolol. (**F**) Norepinephrine and propranolol. (**G**) NNK, norepinephrine and propranolol. (**H**) H_2_O_2_ (positive control). (**I**) H_2_O_2_, NNK or norepinephrine increased DNA damage of NOK-SI cells. These effects were inhibited by the beta-adrenergic antagonist propranolol. Combined treatment of NNK and norepinephrine did not induce significant genotoxic effects. Cortisol enhanced the DNA damage of oral keratinocytes in the presence or absence of NNK. (**J**) Control. (**K**) Cortisol. (**L**) NNK and cortisol. (**M**) Cortisol e RU486. (**N**) NNK, cortisol and RU486. (**O**) H_2_O_2_ (positive control). (**P**) H_2_O_2_, cortisol alone and glucocorticoid associated with NNK increased the DNA damage of NOK-SI cells. Glucocorticoid receptor antagonist RU486 reduced partially and totally the DNA damage induced by the cortisol alone and glucocorticoid combined with NNK, respectively. The results are expressed as the mean ± standard deviation (SD). Upper- and lower cases equal letters indicate a statistically significant difference (*p* < 0.0001). H_2_O_2_ = hydrogen peroxide. NNK = 4 (N-metil-N-nitrosamine)-1-(3-piridil)-butano-1-one). NE = norepinephrine. PROP = propranolol. CORT = cortisol. NNK, NE, PROP and RU486 were used at 10 µM. CORT was used at 100 nM. NOK-SI cells were exposed to NNK and/or stress hormones in the presence or absence of their antagonists for 72 h.

The effects of NNK and stress hormones on single-strand breaks and alkali-label side breaks in the DNA of NOK-SI cells were assessed using the comet assay. After 72 h of exposure, cells treated with NNK or norepinephrine showed an increased DNA damage compared to untreated cells (*p* < 0.0001; Fig. 3B, C, I). These effects were abolished by the beta-adrenergic antagonist propranolol (*p* < 0.0001; Fig. 3E, F, I). Treatment with norepinephrine associated to NNK did not induce significant DNA damage of NOK-SI cells (*p* > 0.05; Fig. 3D, I). Furthermore, an increased tail DNA percentage was observed in cells treated with cortisol alone and cortisol associated with NNK when compared to control cells (*p* < 0.0001; Fig. 3K, L, P). Pre-treatment of NOK-SI cells with glucocorticoid receptor antagonist RU486 inhibited partially and totally the genotoxic effects of cortisol alone and glucocorticoid combined with NNK, respectively (*p* > 0.05; *p* < 0.0001; Fig. 3M, N, P).

### DNA damage induced by tobacco-derived nitrosamine NNK and cortisol in oral keratinocytes is associated with intracellular γH2AX accumulation

**Figure 4:**
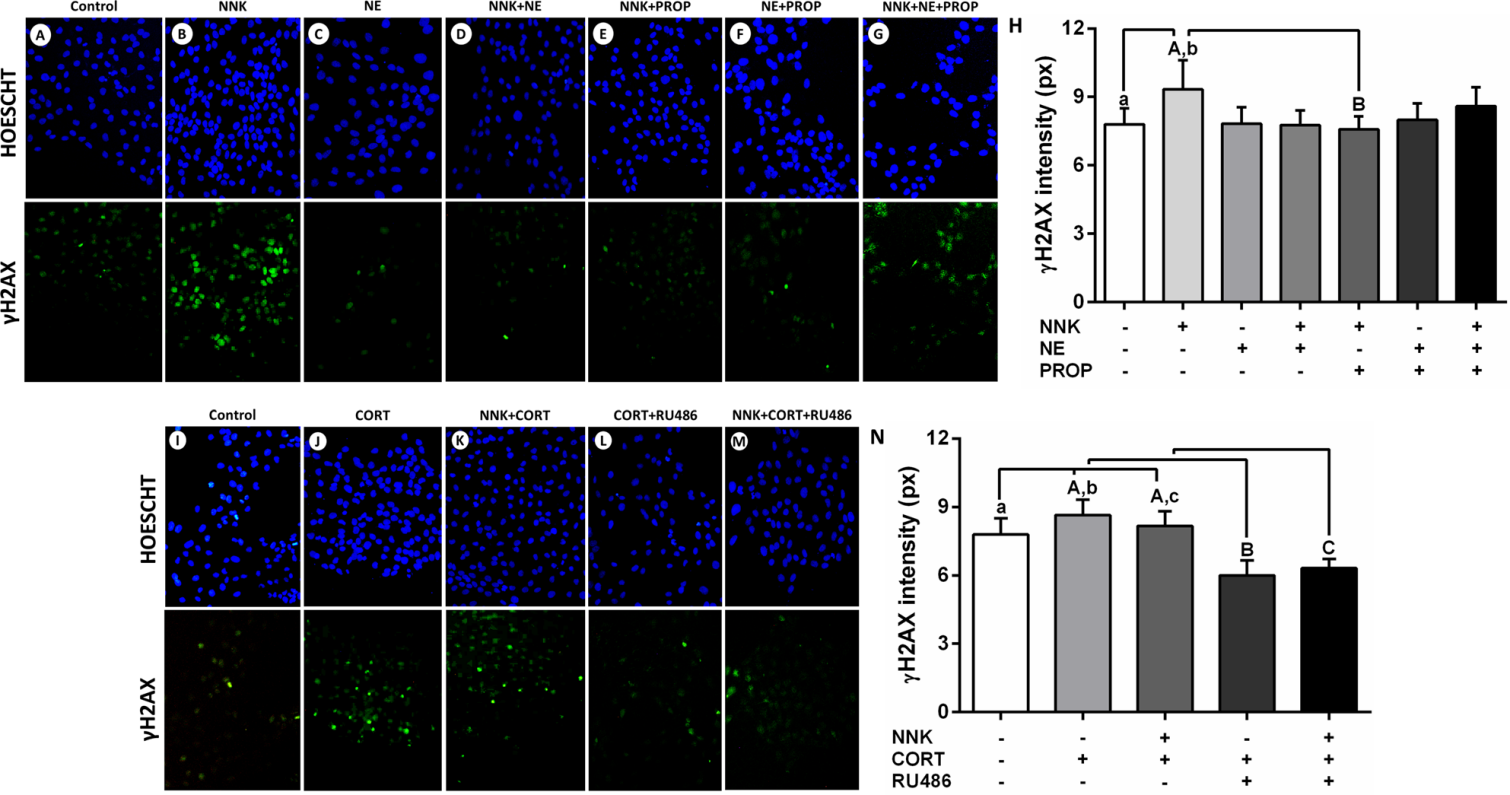
Exposure to the NNK and norepinephrine and γH2AX nuclear expression in oral keratinocytes. (**A**) Control. (**B**) NNK. (**C**) Norepinephrine. (**D**) NNK and norepinephrine. (**E**) NNK and propranolol. (**F**) Norepinephrine and propranolol. (**G**) NNK, norepinephrine and propranolol. (**H**) NNK increased the γH2AX nuclear expression levels. Beta-blocker propranolol inhibited the increased DNA damage caused by the carcinogen. Cortisol increased the γH2AX nuclear expression in oral epithelial cells. (**I**) Control. (**J**) Cortisol. (**K**) NNK and cortisol. (**L**) Cortisol and RU486. (**M**) NNK, cortisol and RU486. (**N**) Cortisol increased the γH2AX nuclear expression levels in the presence or absence of carcinogen NNK. Glucocorticoid receptor blocker RU486 inhibited the increased DNA damage caused by the hormone. The results are expressed as the mean ± standard deviation (SD). Upper- and lower cases equal letters indicate a statistically significant difference (*p* < 0.0001). NNK = 4 (N-metil-N-nitrosamine)-1-(3-piridil)-butano-1-one). NE = norepinephrine. PRO*p* = propranolol. CORT = cortisol. NNK, NE, PROP and RU486 were used at 10 µM. CORT was used at 100 nM. NOK-SI cells were exposed to NNK and/or stress hormones in the presence or absence of their antagonists for 72 h.

Immunofluorescence assays were performed to assess the γH2AX cellular accumulation levels in NOK-SI cells stimulated with NNK and stress hormones. After 4 h of stimulation, cells treated with NNK displayed an increase in the γH2AX accumulation when compared to untreated cells (*p* < 0.0001; Fig. 4B, H). The NNK-induced γH2AX accumulation was inhibited by the pre-incubation with beta-adrenergic antagonist propranolol (*p* < 0.0001; Fig. 4E, H). NOK-SI cells stimulated only with norepinephrine did not display significant changes in the γH2AX accumulation when compared to control cells (*p* > 0.05; Fig. 4C, H). On the other hand, cells treated only with cortisol or hormone combined with NNK showed a higher γH2AX accumulation when compared to untreated cells (*p* < 0.0001; Fig. 4J, K, N). Pre-incubation with glucocorticoid receptor antagonist RU486 inhibited these genotoxic effects (*p* < 0.0001; Fig. 4L, M, N).

### Tobacco-specific carcinogen NNK induces the oxidation of basis in the DNA of oral epithelial cells

**Figure 5:**
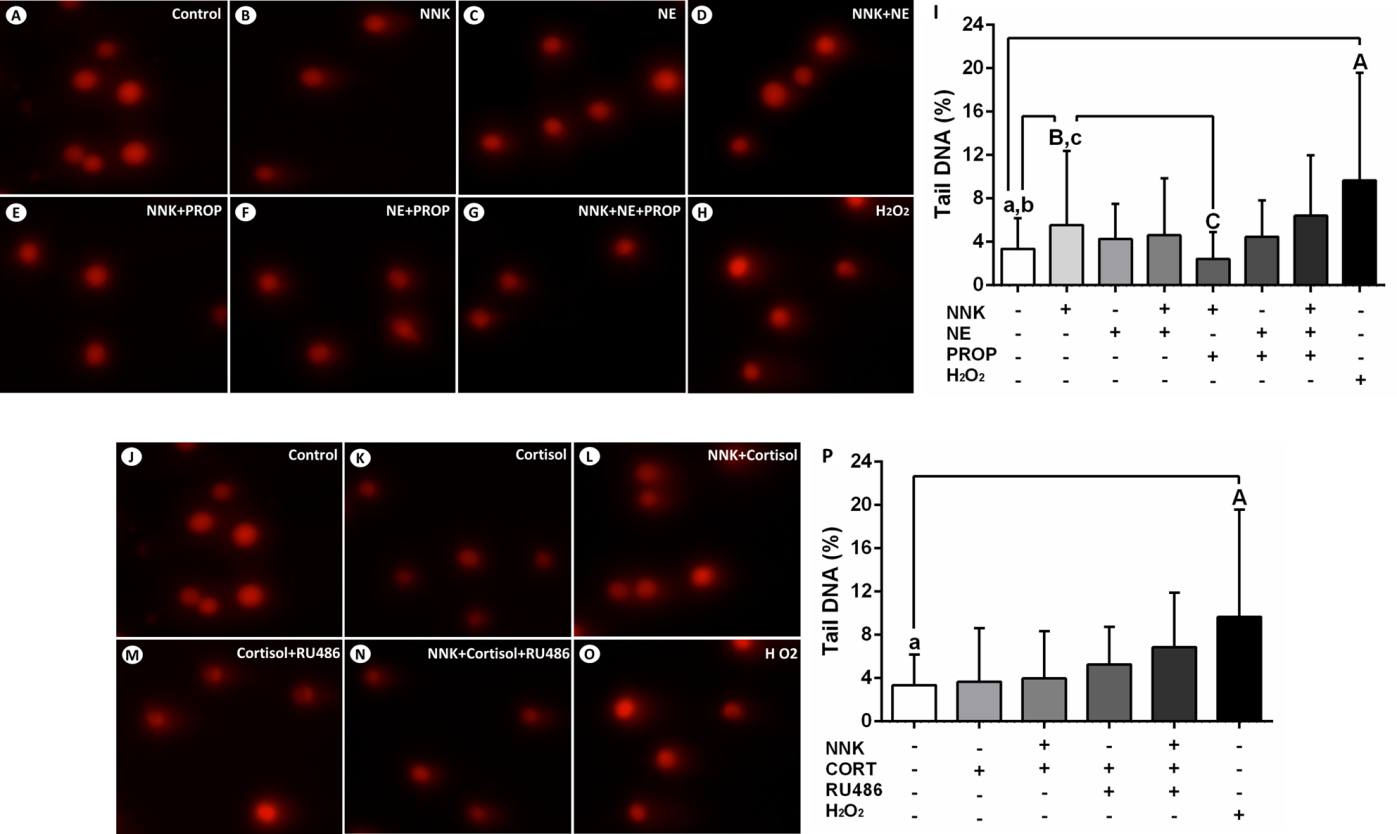
NNK promotes the oxidation of basis in the DNA of NOK-SI cells. (**A**) Control. (**B**) NNK. (**C**) Norepinephrine. (**D**) NNK and norepinephrine. (**E**) NNK and propranolol. (**F**) Norepinephrine and propranolol. (**G**) NNK, norepinephrine and propranolol. (**H**) H_2_O_2_ (positive control). (**I**) Cells treated with H_2_O_2_ or NNK exhibited an increase in the DNA damage, when compared to untreated cells after exposure to FPG enzyme. Beta-adrenergic antagonist propranolol blocked this effect. Norepinephrine in the presence or absence of NNK did not induce significant oxidation of basis. Cortisol did not promote significant oxidative DNA damage. (**J**) Control. (**K**) Cortisol. (**L**) NNK and cortisol. (**M**) Cortisol e RU486. (**N**) NNK, cortisol and RU486. (**O**) H_2_O_2_ (positive control). (**P**) H_2_O_2_ promoted DNA damage in oral keratinocytes. Cells treated with cortisol alone or combined with NNK did not show significant changes regarding the oxidation of basis. The results are expressed as the mean ± standard deviation (SD). Upper- and lower cases equal letters indicate a statistically significant difference (*p* < 0.0001). H_2_O_2_ = hydrogen peroxide. NNK = 4 (N-metil-N-nitrosamine)-1-(3-piridil)-butano-1-one). NE = norepinephrine. PRO*p* = propranolol. CORT = cortisol. NNK, NE, PROP and RU486 were used at 10 µM. CORT was used at 100 nM. NOK-SI cells were exposed to NNK and/or stress hormones in the presence or absence of their antagonists for 72 h.

To evaluate the oxidation of basis induced by the carcinogen NNK and stress hormones in the DNA of NOK-SI cells, FPG-sensitive sites were identified by modified comet assay. After 72 h of treatment, cells stimulated with NNK displayed an increase in the tail DNA percentage when compared to untreated cells (*p* < 0.0001; Fig. 5B, I). This effect was abolished by the beta-adrenergic antagonist propranolol (*p* < 0.0001; Fig. 5E, I). Norepinephrine alone or combined with NNK did not induce significant oxidation of basis compared to control (*p* > 0.05; Fig. 5C, D, I). Cells treated with cortisol in the presence or absence of NNK did not show significant changes regarding the oxidative damage (*p* > 0.05; Fig. 5K, L, P).

### Norepinephrine increases the 8OHdG levels in the presence or absence of carcinogen NNK in oral keratinocytes

**Figure 6:**
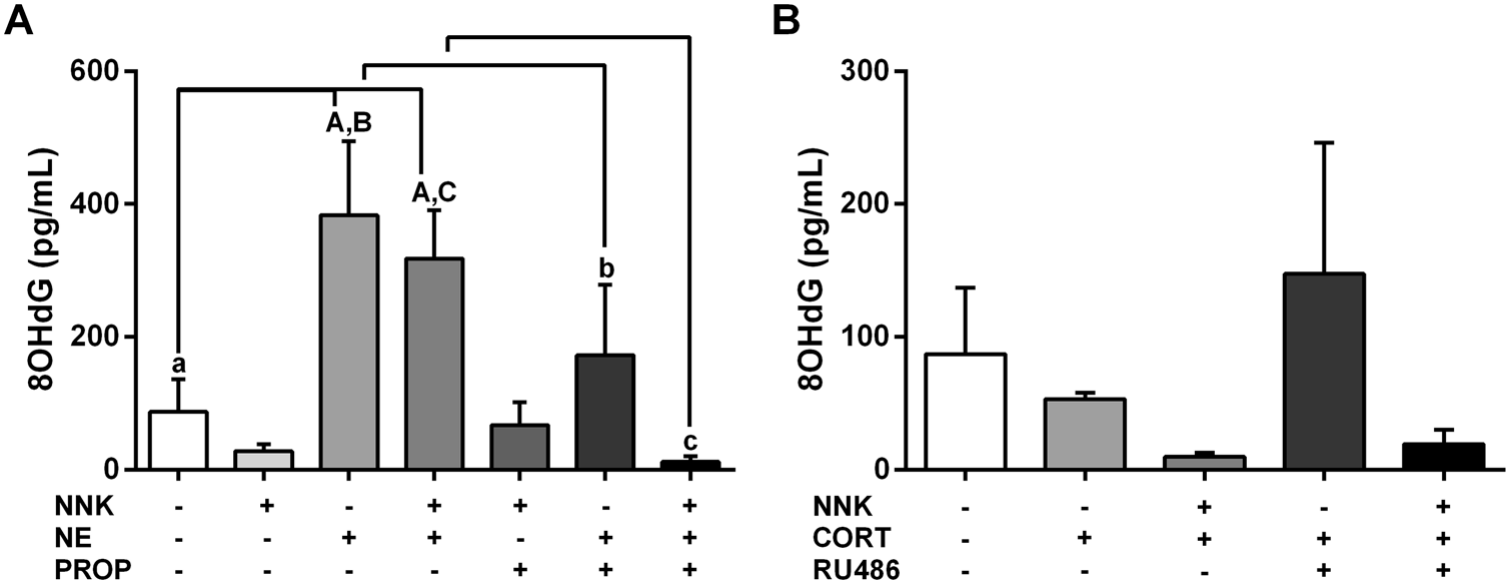
Effects of stress hormones on oxidative DNA damage in oral epithelial cells. (**A**) Norepinephrine up-regulated the 8OHdG levels in the presence or absence of carcinogen NNK. Beta-blocker propranolol inhibited the increased oxidative DNA damage caused by the hormone combined to NNK. (**B**) Cortisol did not change the 8OHdG levels secreted by the NOK-SI cells. The results are expressed as the mean ± standard deviation (SD). Upper- and lower cases equal letters indicate a statistically significant difference (*p* < 0.05). NNK = 4 (N-metil-N-nitrosamine)-1-(3-piridil)-butano-1-one). NE = norepinefrina. PRO*p* = propranolol. CORT = cortisol. NNK, NE, PROP and RU486 were used at 10 µM. CORT was used at 100 nM. NOK-SI cells were exposed to NNK and/or stress hormones in the presence or absence of their antagonists for 72 h.

To assess oxidative DNA damage levels induced by stress hormones in NOK-SI cells, the 8OHdG concentrations were measured in the cell culture medium. After 4 h of treatment, cells stimulated only with norepinephrine or hormone associated to the carcinogen NNK secreted higher 8OHdG levels in the culture supernatant compared to untreated cells (*p* < 0.0001; Fig. 6A). Treatment with the hormone alone resulted in an approximately four-fold increase in the 8OHdG levels. The effects of norepinephrine alone or combined with NNK on the 8OHdG levels were abolished partially and totally by the beta-adrenergic antagonist propranolol, respectively (*p* < 0.05; *p* < 0.001; Fig. 6A). NOK-SI cells treated with carcinogen NNK showed no significant changes in the 8OHdG levels when compared to untreated cells (*p* > 0.05; Fig. 6A). Cortisol did not change the 8OHdG levels secreted by the oral keratinocytes when compared to non-stimulated cells (*p* > 0.05; Fig. 6B).

## Discussion

Investigations have showed that stress hormones may act on oral cancer promoting tumor cell proliferation and invasion^17–20^. Although our previous studies have suggested that psychological stress and related hormones may predict oral carcinogenesis in a preclinical model^18,21^, the mechanisms associated with stress-induced malignant transformation of oral epithelial cells were unknown. In the current study, we used the NOK-SI cell line to confirm the hypothesis that stress hormones and tobacco-specific nitrosamine NNK promote DNA damage and reduce the apoptotic activity of human oral keratinocytes. Our results demonstrated that norepinephrine acted on beta-adrenergic receptors while cortisol activated the glucocorticoid receptor to produce tumorigenic effects in the NOK-SI cells. In this context, we showed the genotoxic action of stress hormones in the presence or absence of NNK, which has not yet been demonstrated in oral keratinocytes.

The role of stress hormones on the promotion of malignant phenotype in normal cells has been increasingly investigated in recent years^2–4,7,22–26^. In these cells, DNA damage is considered a crucial event to trigger the malignant transformation^2,5^. After several cell divisions, an accumulation of damaged DNA may result in irreversible genome mutations commonly found in tumor cells^2^. In the current study, norepinephrine induced an expressive DNA damage of human oral keratinocytes. Similarly, some evidence also show that this adrenergic hormone has also genotoxic effects in normal cells, which may contribute to the tumorigenesis. Flint et al.^4^ demonstrated that norepinephrine at stress concentrations promoted DNA damage in mouse fibroblasts and this effect was abolished by using beta-adrenergic blocker propranolol. Likewise, we induced DNA damage of oral epithelial cells after norepinephrine exposure and reversed this effect with the beta-adrenergic blocker use. Some signaling pathways by which norepinephrine could be promoting DNA damage have been suggested. In a study, norepinephrine treatment promoted DNA damage of normal and cancer cells via PI3K/AKT/MDM2/p53 signaling pathway^7^. Sun et al.^22^ also demonstrated that exposure to norepinephrine can induce DNA damage of embryonic stem cells through the activation of cyclic 3′-5′ adenosine monophosphate (cAMP) and protein kinase A (PKA), molecules that constitute the cAMP/PKA pathway. Similarly to our study, these investigations also used norepinephrine at 10 µM to produce an increased DNA damage, which may be considered a high concentration that simulate chronic stress conditions. We previously demonstrated that norepinephrine at 10 µM enhanced the proliferation of oral cancer cells^19^.

Activation of the beta-adrenergic receptors by the norepinephrine can result in the upregulation of cAMP/PKA pathway, which increases mitochondrial oxidative phosphorylation and reactive oxygen species (ROS) production^2,27^. ROS may damage mitochondrial and nuclear DNA and produce a wide range of DNA lesions such as strand breaks, thymine glycol, base loss and base damage including 8OHdG^2^. In the current study, norepinephrine induced single-strand breaks and alkali-label side breaks in the DNA of NOK-SI cells. In addition, adrenergic hormone in the presence or absence of NNK also promoted oxidative DNA damage resulting in a higher secretion of 8OHdG levels. This event was dependent of beta-adrenergic receptors. Another investigation found higher 8OHdG cellular levels in the DNA genomic of cardiac myoblasts after exposure to norepinephrine^28^. 8OHdG has been considered the most common mutagenic biomarker associated to oxidative DNA damage^2^. It is closely related to the development of several types of cancer in humans and has also been used to estimate the DNA damage after exposure to carcinogenic agents^29^. In oral cancer patients, elevated salivary 8OHdG levels have already been related to tumor progression and also seem to be involved in the pathogenesis of the disease^30,31^. The increased 8OHdG immunoexpression in non-tumor tissues contribute to the chemical oral carcinogenesis in preclinical models^32,33^.

In the current study, norepinephrine combined to nitrosamine NNK inhibited the apoptosis of NOK-SI cells by decreasing the activity of caspases 3 and 7. This inhibition would permit an uncontrolled proliferation of keratinocytes with damaged DNA, whose tumorigenic mutations could lead to the oral cancer development. The reduced expression of caspase 3 in oral cancer tissues has been related to malignant transformation^34^. A recent investigation demonstrated that norepinephrine protected against apoptosis of mesenchymal stem cells by inhibiting the expression of caspase 3 via AKT/BCL-2^11^. It has also been suggested that norepinephrine regulates cell apoptosis through the activation of cAMP/PKA pathway via beta-adrenergic receptors^35^. There is substantial evidence that norepinephrine released from circulating blood and local sympathetic nerve fibers bind to beta-adrenergic receptors on the cell membrane and induce the synthesis of cAMP, responsible for triggering oncogenic mechanisms^35^. cAMP activates PKA that phosphorylates several proteins including transcription factors of the ATF/CREB and GATA families^35,36^. Moreover, cAMP-induced up-regulation of Exchange Protein activated by Adenylyl Cyclase (EPAC) triggers the Ras/Raf/ MEK/ERK pathway regulating the transcription factors of the AP1 and ETS families^35,37^. In malignant cells, all these mechanisms mediated by cAMP influence the production of cytokines and growth factors, as well as cell morphology, motility and proliferation^35^. Beta-adrenergic signaling activated by the norepinephrine may also up-regulate the phosphorylation of the transcription factor STAT3, which modulates tumorigenic pathways^38^.

In epithelial cells of the upper aerodigestive tract, DNA damage promoted by tobacco-specific carcinogenic substances has been considered a crucial event for malignant transformation^5^. In the current study, NOK-SI cells were treated with the tobacco-specific nitrosamine NNK at a tumorigenic concentration commonly found in chronic smokers^39^. Although TUNEL assay has shown that nitrosamine did not induce significant fragmentation of the acid nucleic in these cells, comet assay demonstrated that NNK promotes single-strand breaks and alkali-label side breaks in the DNA of NOK-SI cells. In addition, NNK also induced the oxidation of basis in the presence of FPG. All genotoxic effects were mediated via beta-adrenergic receptors. These findings suggest that a long period of exposure to NNK would be needed to promote the induction of malignant phenotype in these cells. In bronchial epithelial cells, Shen et al.^40^, for example, induced a malignant phenotype after 192 h of exposure to nitrosamine. In our study, TUNEL assay showed that NNK almost inhibited the genotoxic effect caused by the norepinephrine while comet assay demonstrated that chemical carcinogen in the presence of norepinephrine did not promote significant DNA damage. This can be explained due to a decrease in the sensitivity of the beta-adrenergic receptors that may be downregulated when they are exposed to several agonists, such as catecholamines and NNK^6^. In human cells, NNK is enzymatically converted into metabolites, which bind to the DNA molecule and induce tumorigenic mutations^5,6^. These genotoxic effects can be mediated by nicotinic acetylcholine and beta-adrenergic receptors^6^. Although the tumorigenic action of nicotinic receptors induced by the NNK binding has been demonstrated in oral epithelial cells^41–44^, the role of beta-adrenergic signaling activated by the nitrosamine still need to be investigated. In other types of normal epithelial cells^45,46^, the tumorigenic action of beta-adrenergic receptors induced by NNK binding has already been evaluated. Beta-adrenergic receptors activated by the nitrosamine may mediate, for example, the levels of ERK1/2 MAP kinases in mammary cells^45^. In small airway cells, beta-adrenergic signaling activation by NNK can up-regulate ERK1/2 and ATF1/CREB phosphorylation via PKA^46^.

Cortisol secreted into the circulation binds to the glucocorticoid receptor in the cytoplasm and translocate it to the nucleus where they modulate the transcription of several genes by associating to DNA response elements and/or to other transcription factors^47^. Cortisol may lead to phosphorylation of MDM2 that down-regulates the function of the tumor suppression protein p53, mediator responsible for regulating several mechanisms associated to the DNA repair^2,25^. The persistent accumulation of DNA damage enhances the risk of malignant transformation predisposing the tumor cell development^2^. These findings concerning the MDM2/p53 signaling pathway were demonstrated in mouse embryonic fibroblasts after treatment of the cells with corticosterone, a glucocorticoid similar to cortisol found in mice and rats^25^. Another investigation showed that cortisol at 1000 nM, considered a pharmacological concentration, promoted approximately a four to five-fold increase in DNA damage of 3T3 mouse fibroblasts after 10 min of exposure to the hormone, and this effect was reversed using antagonist RU486^3^. Our results showed that cortisol at 100 nM promoted single-strand breaks and alkali-label side breaks in the DNA of oral epithelial cells and these effects were inhibited partially and totally, by the glucocorticoid receptor antagonist RU486 in the absence or presence of NNK, respectively. In the presence of FPG, cortisol did not induce significant DNA damage in NOK-SI cells. These results suggest that cortisol may not be acting only on glucocorticoid receptors. The induction of DNA damage in oral keratinocytes could be modulated by the activation of other mechanisms triggered by the glucocorticoid, including those mediated by the mineralocorticoid receptors.

The current study showed that a higher γH2AX cellular accumulation was detected after exposure to NNK and/or glucocorticoid, and this event was completely reversed by RU486 treatment. In the current study, the concentration designed for cortisol simulated chronic stress conditions^19^. Recent studies have shown that cortisol exposure also promote an accumulation of γH2AX in breast cancer cells^8,48,49^. In these investigations, the analysis of γH2AX phosphorylation was used as an indicator of DNA damage induced by the cortisol, whose effects were blocked with RU486 use^8,48,49^. These findings suggest that cortisol produces genotoxic effects mediated via glucocorticoid receptor. Currently, there is a lack of consensus regarding the cortisol concentration to induce these effects in normal and tumor cells. The quantification of γH2AX levels can also be used to determinate the DNA lesions resulting from exposure to environmental carcinogens such as nitrosamines^10,50^. In our results, NNK promoted an increase in the γH2AX nuclear expression in oral keratinocytes and this genotoxic effect was abrogated by the beta-adrenergic blockade with propranolol. In oral carcinogenesis, tissue γH2AX immunoexpression increases significantly before malignant transformation being considered an indicator for oral cancer development^51^. In the TUNEL assay, NNK inhibited the DNA fragmentation induced by the cortisol. A rapid metabolization of the glucocorticoid in the presence of NNK could have avoid this more severe genotoxic effect. The enzyme 11β-Hydroxysteroid dehydrogenase type 1 (11β-HSD 1), which is involved in the metabolism of cortisol can also metabolize the tobacco-specific nitrosamine NNK^52^. The presence of NNK in the culture medium could have increased the production of 11β-HSD 1 in the NOK-SI cells and consequently the consumption of cortisol.

The current study shows that cortisol promotes anti-apoptotic effects in NOK-SI cells by reducing the activity of caspases 3 and 7. In addition to DNA damage, these effects induced by the glucocorticoid could contribute to a proliferative malignant behavior of the oral keratinocytes. Glucocorticoid receptor antagonist RU486 did not reverse the anti-apoptotic effects induced by the cortisol. Other mechanisms triggered by the glucocorticoid could be modulating the activity of caspases 3 and 7 in the oral keratinocytes, including the activation of mineralocorticoid receptors. In breast cancer cell lines, dexamethasone (a glucocorticoid) at 1000 nM inhibited chemotherapy-induced apoptosis and promoted the gene expression of SGK1 and MKP1, whose proteins are associated to cell survival^12^. After glucocorticoid exposure, proapoptotic genes, caspases 3, 8 and 9 and pro-apoptotic BCL-2 family members were downregulated^12^. In another study, dexamethasone inhibited the activity of caspases in lung and cervical cancer cell lines preventing therapy-induced tumor reduction in orthotopic model^53^. Similarly to these findings, the current study demonstrates that cortisol in a stress concentration may affect cell death of normal oral keratinocytes. These cells might then proliferate continuously with damaged DNA becoming malignant.

This study showed that stress hormones may induce DNA damage of oral keratinocytes. In our experiments, we have succeed in evaluating the genotoxic role of each hormone in the presence or absence of NNK, which is not possible to verify clinically. Although this study has brought advances regarding the use of in vitro model to better understand the role of stress hormones on the initial steps of oral carcinogenesis process in humans, it has also some limitations that need to be considered. It was not possible to determine the minimal amount of damaged DNA responsible to induce the biological responses. In addition, the dynamic binding between stress hormones and their receptors as well as the regulation of signaling pathways by the beta-adrenergic receptors in the presence of NNK and/or norepinephrine were not evaluated in the current study. The cells were also not tested with different concentrations of cortisol once it seems to have dual effects on tumorigenic processes, as we previously reported^18,19^. From the results of the current study, the genotoxic effects of stress hormones could be investigated in primary cultures or other oral keratinocytes cell lines.

To date, no study has evaluated the genotoxic effects of stress hormones associated to tobacco-specific nitrosamines on the promotion of genomic lesions in normal oral keratinocytes. Our findings show that high concentrations of norepinephrine and cortisol that simulate chronic stress cause expressive DNA damage of NOK-SI cells, predisposing them to malignant transformation. The occurrence of this event may be confirmed by the evaluation of the increased cellular levels of biomarkers associated to the DNA damage. The genotoxic effects caused by the stress hormones in epithelial cells could increase the risk of developing oral cancer.

## Material and methods

### Cells and culture conditions

Normal Oral Keratinocyte-Spontaneously Immortalized (NOK-SI) cell line was kindly provided by the Dr. Aline Satie Takamiya (São Paulo State University—UNESP, Araçatuba, São Paulo, Brazil). NOK-SI cells were cultured in Dulbecco Modified Eagle Medium (DMEM; Gibco, Carlsbad, CA, EUA) containing 4 mM l-glutamine, 1.5 g/L sodium bicarbonate and 4.5 g/L glucose. All reagents used in this study were purchased from Sigma Aldrich (St. Louis, MO, USA). The culture medium was supplemented with 10% fetal bovine serum (FBS), 100 μg/mL streptomycin, 100 μg/mL penicillin and 0.1% gentamicin. NOK-SI cells were grown in 75-cm^2^ culture flasks (Greiner Bio-One, Kremsmünster, Austria) at 37 °C in humidified atmosphere of 5% (v/v) CO_2_. After reaching approximately 70% cell density, the cells were trypsinized, seeded in 6-well plates and then maintained under the same conditions. In these plates, NOK-SI cells were stimulated with NNK (CASNr 64091-91-4) and/or stress hormones, norepinephrine (CASNr 3414-63-9) or cortisol (CASNr 50-23-7), after reaching about 50% cell density. All reagents were purchased from Merck (Merck KGaA, Darmstadt, Germany).

### Carcinogen and hormone treatment

The growth medium was replaced with NNK and/or norepinephrine at 10 µM or cortisol at 100 nM, according to previous studies^19,53^. These concentrations simulate chronic stress conditions in humans. NNK and stress hormones were added at the same time in the groups which carcinogen was in the presence of norepinephrine or cortisol. The blocking of hormone receptors was performed by pre-incubating cells with beta-adrenergic receptor antagonist propranolol and glucocorticoid receptor antagonist RU486. Both antagonists were diluted in the culture medium to achieve the concentration of 10 µM. Cells were incubated in the presence of antagonists for 1 h prior to the addition of NNK and/or norepinephrine or cortisol. NOK-SI cells were cultured without FBS overnight and then exposed to the NNK and/or hormone for 4 h in culture medium without FBS. Moreover, cells were also cultured with 10% FBS and stimulated with carcinogen and/or hormone for 72 h in a culture medium with 10% FBS. In this case, the stimuli were replaced every 24 h along with the renewal of the culture medium. Preliminary experiments showed that the suppression of FBS for 72 h, even in the presence of stress hormones, promoted morphological changes and high mortality rate in NOK-SI cells.

### Terminal deoxynucleotidyl transferase dUTP nick-end labelling (TUNEL assay)

The cells were washed in phosphate-buffered saline solution (PBS) with 1 mg/mL of polyvinyl alcohol (PBS/PVA) and fixed using a 4% paraformaldehyde solution for 1 h at room temperature. Then, NOK-SI cells were permeabilized using 0.5% Triton X-100 for 30 min and washed with PBS/PVA. For the positive control, untreated cells were incubated with DNAse I (50 IU/mL) for 1 h at room temperature while cells from the experimental groups were maintained in PBS/PVA. Cells were incubated in 30 µL of TUNEL reaction mixture (In-Situ Cell Death Detection Kit (Roche Diagnostics Corp., Indianapolis, IN, USA) for 1 h at room temperature, according to the manufacturer’s recommendations. For the negative control, untreated cells were incubated with label solution only. The cells were washed in PBS/PVA and stained with Hoechst 33342 (1 µg/mL) for 10 min at room temperature. Then, they were washed again in PBS/PVA and transferred to the glass slides using the Vectashield mounting medium (Vector Laboratories, Burlingame, CA, USA). Coverslips were placed. The cells were analyzed using an inverted epifluorescence microscope (Olympus America Inc, Center Valley, PA, USA). The percentage of TUNEL-positive cells showing fragmented DNA was then determined by a blinded researcher.

### Activity of caspases-3 and -7

The activity of caspases-3 and -7 was measured in the NOK-SI cells using the Image-iT LIVE Red Caspase-3 and -7 Detection Kit (Molecular Probes, Invitrogen, OR, USA) according to the manufacturer’s instructions. The cells were incubated in 10-µL of a fluorescent inhibitor of caspases-3 and 7 for 1 h at 37 °C in a humid chamber. Cells were washed with PBS buffer solution and fixed in 4% paraformaldehyde solution for 40 min at room temperature. The cells were then stained with Hoechst 33,342 (1 µg/mL) and the slides mounted with Vectashield (Vector Laboratories, Burlingame, CA, USA). Stained cells were photographed using an inverted microscope (Olympus America Inc, Center Valley, PA, USA). A blinded researcher to the experimental groups quantified the fluorescence signal intensities (pixels) of the stained cells in the recorded images running the ImageJ software (National Institutes of Health, Bethesda, MD, USA).

### Single cell gel electrophoresis (comet assay)

Adherent cells were trypsinized from the plates, mixed in 0.5% low-melting agarose and placed in glass slides pre-coated with 1% agarose gel. The slides were immersed in lysis solution (1 M Tris, 1 M NaCl, 1 M Na2EDTA, 1% Triton X-100) with proteinase K for 2 h at 50 °C. Then, they underwent electrophoresis at 25 V for 30 min and neutralized with 0.4 M Tris–HCl for 5 min. The cells were then stained with ethidium bromide (2 μg/mL) and photographed using an inverted microscope (Olympus America Inc, Center Valley, PA, USA) with an excitation wavelength of 495 nm and an emission wavelength of 520 nm. In each group, a total of 100 cells was analyzed. The percentage of DNA in the comet tail was determined using the CASP software (University of Wroclaw, Wroclaw, Poland) by a blinded researcher to the experimental groups.

### FPG (DNA-formamidopyrimidine glycosylase)-modified comet assay

As described above, cells were firstly mixed in low-melting agarose, placed in glass slides pre-coated with agarose gel and immersed in lysis solution. The slides were washed in the FPG buffer (40 mM HEPES, 0.1 M KCl, 0.5 mM EDTA, pH 8) and incubated with the enzyme (1:3000) or buffer for 45 min at 37 °C. Then, they were subjected to electrophoresis, neutralized, stained with ethidium bromide, photographed and analyzed as described for the conventional comet assay. The level of FPG-sensitive sites was calculated as the difference in percentage (%) of tail DNA between FPG-treated cells and control cells (not-treated with the enzyme).

### Immunoexpression of phospho-H2AX (γH2AX)

Cells were fixed with 4% paraformaldehyde solution in glass-bottom tissue culture plates and maintained in methanol for 20 min at 4 °C. Non-specific binding sites were then blocked by using 1% bovine serum albumin (BSA) for 2 h at room temperature. Cells were incubated overnight at 4 °C with primary antibody for anti-phospho-H2AX (1:200; Cell Signaling Technology, Beverly, MA, USA). Then, the secondary antibody was incubated for 1 h at room temperature. The cells were stained with Hoechst 33342 (1 µg/mL) and the slides mounted with Vectashield (Vector Laboratories, Burlingame, CA, USA). NOK-SI cells were photographed using an inverted microscope (Olympus America Inc, Center Valley, PA, USA) and a blinded researcher to the experimental groups quantified the fluorescence signal intensities (pixels) of the stained cells in the recorded images by using the ImageJ software (National Institutes of Health, Bethesda, MD, USA).

### Measurement of 8-hydroxy-2′-deoxyguanosine (8OHdG) content

The 8-hydroxy-2′-deoxyguanosine (8OHdG) levels were measured in the culture medium using a specific kit (DNA/RNA Oxidative Damage; Cayman Chemical, Michigan, USA) by the method Enzyme-Linked Immunosorbent Assay (ELISA). The ELISA assay sensitivity was 30 pg/mL for 8OHdG. This assay was performed in triplicate according to the manufacturer’s recommendations.

### Statistical analysis

GraphPad Prism 8.21 (GraphPad Software Inc., San Diego, CA, USA) was used to perform statistical analysis. The one-way analysis of variance (ANOVA) with post-hoc Tukey test for multiple comparisons evaluated possible differences between the groups. The results were presented as mean ± standard deviation (SD). Differences were considered statistically significant when *p* < 0.05.

## Data Availability

The datasets generated during and/or analyzed during the current study are available from the corresponding author on reasonable request.
